# VH-4-A Bioactive Peptide from Soybean and Exercise Training Constrict Hypertension in Rats through Activating Cell Survival and AMPKα1, Sirt1, PGC1α, and FoX3α

**DOI:** 10.3390/molecules27227705

**Published:** 2022-11-09

**Authors:** Rathinasamy Baskaran, Balamuralikrishnan Balasubramanian, Jou-Hsuan Ho, Ming-Fu Wang, Mosleh Mohammad Abomughaid, Hong-Siang Yang, Wan-Teng Lin

**Affiliations:** 1 Department of Bioinformatics and Medical Engineering, Asia University, Taichung 413305, Taiwan; 2 Department of Food Science and Biotechnology, College of Life Science, Sejong University, Seoul 05006, Korea; 3 Department of Food Science, Tunghai University, Taichung 407224, Taiwan; 4 Department of Food and Nutrition, Providence University, Taichung 43301, Taiwan; 5 Department of Medical Laboratory Sciences, College of Applied Medical Sciences, University of Bisha, Bisha 61922, Saudi Arabia; 6 Department of Hospitality Management, College of Agriculture, Tunghai University, Taichung 407224, Taiwan

**Keywords:** soybean peptides, swimming exercise, spontaneous hypertension rats, hypertension, AMPK

## Abstract

Hypertension is a chronic disease related to age, which affects tens of millions of people around the world. It is an important risk factor that causes myocardial infarction, heart failure, stroke, and kidney damage. Bioactive peptide VHVV (VH-4) from soybean has shown several biological activities. Physical exercise is a cornerstone of non-pharmacologic treatment for hypertension and has established itself as an effective and complementary strategy for managing hypertension. The present study evaluates the efficacy of VH-4 supplement and swimming exercise training in preventing hypertension in spontaneously hypertensive rats (SHR). SHR animals were treated with VH-4 (25 mg/kg by intraperitoneal administration) and swimming exercise (1 h daily) for eight weeks, and the hemodynamic parameters, histology, and cell survival pathway protein expression were examined. In SHR rats, increased heart weight, blood pressure, and histological aberrations were observed. Cell survival protein p-PI3K and p-AKT and antiapoptosis proteins Bcl2 and Bcl-XL expression decreased in SHR animals. SIRT1 and FOXO3 were decreased in hypertensive rats. Both bioactive peptide VH-4 treatment and swimming exercise training in hypertensive rats increased the cell survival proteins p-PI3K and p-AKT and AMPKα1, Sirt1, PGC1α, and FoX3α proteins. Soy peptide VH-4, along with exercise, acts synergistically and prevents hypertension by activating cell survival and AMPKα1, Sirt1, PGC1α, and FoX3α proteins.

## 1. Introduction

Globally, hypertension is the most significant potential risk factor for both cardiovascular disease (CVD) and increased mortality [1]. Reducing potential hazards such as high sodium consumption, low potassium intake, obesity, alcohol consumption, insufficient physical exercise, and bad eating habits is indicated for the prevention and control of hypertension. Other possible risk factors for hypertension were identified, such as noise exposure, air pollution, mental anguish, and smoking cigarettes [2]. The risk of hypertension has also been correlated to psychosocial stress and alternating work shifts. In low- and middle-income nations, awareness, treatment, and management of hypertension are at obscene levels [3]. Adults with hypertension are now classified by the American College of Cardiology/American Heart Association Task Force on Clinical Practice Guidelines as having a systolic blood pressure of 130 or above and a diastolic blood pressure of 80 or below. This modification was made in response to evidence from several large-scale, prospective observational studies that found a substantial increase in CVD risk when BP increased [4].

The regulation of AMP-activated protein kinase (AMPK) signaling is crucial for regulating cellular homeostasis since AMPK is characterized as a cellular energy sensor [5]. Stressful circumstances and low energy levels trigger the expression of AMPK signaling. Increased calcium accumulation inside the cells phosphorylates AMPK at T172 and activates adenosine monophosphate (AMP) levels independently. This leads AMPK to be upregulated in a Ca^2+^/CaM-dependent protein kinase β (CaMKKβ)-dependent manner [6]. There is a strong correlation between inflammation and AMPK signaling in a number of diseases, including hypertension [7]. One of the most well-known AMPK signaling downstream targets in the inflammatory process is the nuclear factor-κB (NF-κB) [8]. The regulation of glucose metabolism was tightly regulated by AMPK signaling and its downstream targets. Reactive oxygen species (ROS) are one of the key signaling molecules that stimulate AMPK signaling, which then causes peroxisome proliferator-activated receptor gamma coactivator 1-alpha (PGC-1α) upregulation and leads to mitochondrial biogenesis [9]. Importantly, AMPK signaling also triggers sirtuin 1 (SIRT1) expression to increase PGC-1α expression [10].

Enzymatic hydrolysis and fermentation processes can produce bioactive peptides from dietary proteins. The food and pharmaceutical sectors may use these peptides as nutraceuticals to improve health [11]. Both intact and hydrolyzed soy protein are often identified as a major source of nitrogen in formulas for infants and adults [12]. Soybean proteins are known to perform a number of physiological tasks, including decreasing cholesterol and reducing body fat [13]. As a result, during the past few years, the market for functional foods that promote health has increased [14]. The fermentation technique has been used to process enzymes using soy. In enzyme-hydrolyzed soy proteins and fermented soy products, many antihypertensive peptides have been reported [15].

For optimum impact, bioactive peptides need to enter the bloodstream via the gut wall and maintain their activity while being broken down by human proteases. The transport and bioavailability of intact peptides are significantly influenced by their physical characteristics, including hydrophobicity, charge, peptide sequence, and small molecular size [16]. Small peptides are more readily absorbed than lengthy peptides because they might more easily cross the intestinal barrier and function at the cellular level. It has been demonstrated that once bioactive peptides cross the intestinal barrier, a minimal concentration is needed for them to have a specific role at the tissue level [17].

Physical exercise is a cornerstone of non-pharmacologic treatment for hypertension and has established itself as an effective and complementary strategy for managing hypertension. Related research has also extensively substantiated physical exercise’s peripheral effects [18,19]. The antihypertensive benefits of physical exercise, including decreased resting and ambulatory blood pressure and improvements in cardiorespiratory fitness, have been established as helpful strategies for cardiovascular health. The advantages of physical activity are in lowering blood pressure and cardiovascular risk variables, as well as developing fitness levels, body composition, and quality of life, and lowering mortality risk [20].

The research on bioactive peptides and how they work synergistically with exercise might be useful. Using techniques from exercise and nutrition supplements, public health and well-being may be improved and maintained [21]. In this circumstance, preserving or even enhancing pertinent hypertension-related characteristics such as functional capacity and mitochondrial function is the objective. Bioactive peptides and exercise training might thus be another potentially focused and effective approach to dealing with several facets of hypertension. VHVV extracted from soy protein isolate has lipolysis-stimulating properties [22]. Previous studies have shown that VHVV peptides increased long-term memory and renal damage in hypertensive rats [23,24]. In the present study, we elucidate the antihypertensive effect of the VH-4 peptide in the SHR hypertensive model and further establish the synergistic effects of VH-4 with exercise training in alleviating hypertension.

## 2. Results

### 2.1. Effect of Combination of VH-4 Peptide and Exercise on Heart Weight, Blood Pressure, Ejection Fraction (EF), and Fractional Shortening (FS) of SHR Rats

**Figure 1:**
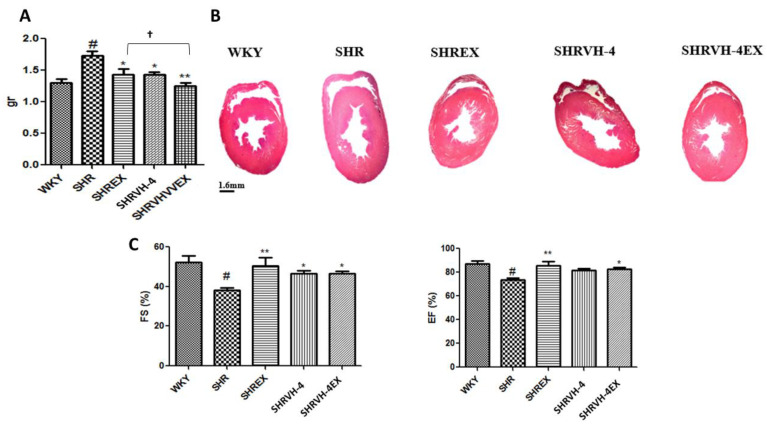
Effect of combination of VH-4 peptide and exercise on heart weight, ejection fraction (EF), and fractional shortening (FS) of SHR rats. (**A**) Heart weight, (**B**) ejection fraction % and fractional shortening %, (**C**) tomography of the cardiac tissue section. # *p* < 0.01 compared to WKY, * *p* < 0.05, ** *p* < 0.01 compared to SHR, and † *p* < 0.05 compared to SHR + EX and/or SHR + VH-4.

In hypertensive rats, heart weight was significantly increased when compared to control WKY, whereas VH-4 treatment and exercise significantly reduced the heart size in SHR animals. The results were confirmed by the tomography of the heart tissue section (Figure 1A,B). On the other hand, electrocardiography (ECG) parameters EF% and FS% were significantly reduced in SHR rats when compared to WKY control. Combine VH-4 treatment and exercise training in SHR rats significantly these EF% and FS% (Figure 1C).

Hemodynamic parameters, such as HR—heart rate, SBP—systolic blood pressure, MBP—mean blood pressure, and DBP—diastolic blood pressure, were determined in the treatment group (Table 1). All these parameters were found to be significantly increased in the SHR group when compared to the control WKY group. VH-4 treatment and swimming exercise significantly increased HR, MBP, and DBP in SHR rats.

### 2.2. Effect of VH-4 and Exercise on Histology of Control and Treatment Groups

**Figure 2:**
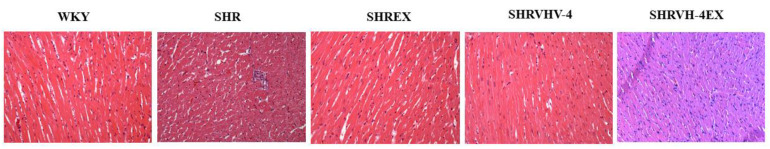
Effect of VH-4 and exercise on heart histology of control and treatment groups. H&E staining. Magnification 100×.

Histological abortion in the hypertensive rats was analyzed by H&E staining (Figure 2). Normal cellular and histological architecture were observed in the WKY control group. In SHR animals, blurred cell edges and severe myofiber disarrangement were observed. However, these histological abnormalities were greatly diminished in the SHR animal, which receives VH-4 peptide treatment and exercise training alone and combined.

### 2.3. Effect of VH-4 and Exercise Training on the Cell Survival Pathway and Apoptosis in the Heart Tissue Section of SHR Animals

**Figure 3:**
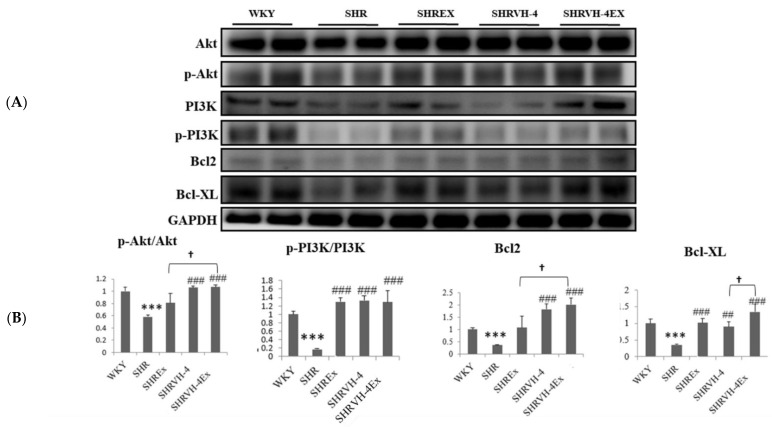
Effect of VH-4 and exercise training on the cell survival pathway and apoptosis in the heart tissue section of SHR animals. (**A**). Western blot of PI3K, AKT, Bcl2, and Bcl-XL. (**B**) Graph showing densitometric analysis. *** *p* < 0.001 compared to WKY, ## *p* < 0.01, ### *p* < 0.001 compared to SHR, and † *p* < 0.05 compared to SHR + EX and/or SHR + VH-4.

To understand the effect of soybean peptide and swimming exercise training on cell survival pathway protein expression, western blot analysis was performed (Figure 3). In hypertensive rats, the expression of cell survival proteins p-AKT and p-PI3K was significantly reduced when compared to the control. Combined VH-4 treatment and exercise training significantly upregulated the p-AKT and p-PI3K expression in SHR animals. The expression of antiapoptotic proteins Bcl2 and Bcl-XL was also significantly reduced in the SHR group, whereas VH-4 treatment and exercise significantly increased the expression of Bcl2 and Bcl-XL. However, combined VH-4 treatment and exercise have a better effect on activating antiapoptotic proteins Bcl2 and Bcl-XL expressions than VH4 treatment or exercise alone in SHR animals.

### 2.4. Effect of VH-4 and Exercise Training on AMPKα1, Sirt1, PGC1α, and FoX3α Signaling Pathway in SHR Animals

**Figure 4:**
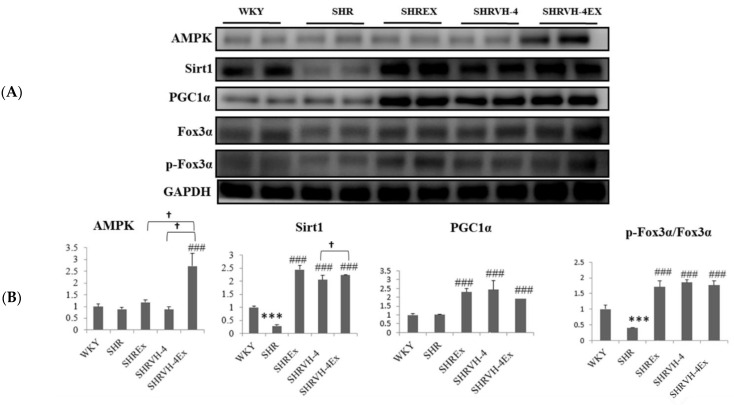
Effect of VH-4 and exercise training on AMPKα1, Sirt1, PGC1α, and FoX3α signaling pathway in SHR animals. (**A**) Western blot of AMPKα1, Sirt1, PGC1α, and FoX3α. (**B**) Graph showing densitometric analysis. *** *p* < 0.001 compared to WKY, ### *p* < 0.001 compared to SHR, and † *p* < 0.05 compared to SHR + EX and/or SHR + VH-4.

AMPKα1, Sirt1, PGC1α, and FoX3α expressions were then quantified (Figure 4). AMPKα1 and PGC1α expression was not significantly altered in SHR animals. Expression of Sirt1 and p-FoX3α was found to be significantly increased in the SHR group, compared to the control WKY group, whereas soy peptide VH-4 and exercise significantly upregulated AMPKα1, Sirt1, PGC1α, and p-FoX3α expression compared to the SHR group. However, combining exercise and DF treatment led to higher significance than VH-4 and exercise alone, suggesting its synergistic effect in inducing the expression of AMPK, SIRT1, PGC-1α, and FOXO3 in SHR animals.

## 3. Discussion

Due to its high morbidity and mortality, hypertension stands out as the single biggest risk factor and is a global issue in terms of public health. The number of hypertension patients has risen steadily in recent decades as a result of an elderly global population and population growth [25]. In addition to causing heart diseases, hypertension also affects other tissues and organs, which leads to renopathy, vasculopathy, and cerebrovascular diseases [26]. Previous studies showed that individuals who have hypertension engaged in less physical activity than those without hypertension [27].

Angiotensin-converting enzyme (ACE), a non-specific dipeptidyl carboxypeptidase, controls the renin-angiotensin system (RAS), which in turn controls blood pressure [28]. Angiotensinogen is broken down by renin, an aspartic protease, into inactive angiotensin I decapeptide, which is subsequently broken down by ACE to release the octapeptide angiotensin II, a potent vasoconstrictor that regulates blood pressure and some hormones [29]. Bradykinin is a nonapeptide that lowers blood pressure, and ACE catalyzes its breakdown in the kallikrein–kinin cycle [30]. Synthetic ACE inhibitors have been demonstrated to be highly efficient in lowering blood pressure; however, there are safety concerns that prevent their widespread use as therapeutics [31]. Thus, using bioactive peptides to prevent the conversion of angiotensin I to angiotensin II can be a useful substitute for hypertension. Among several bioactive peptides, ACE-inhibitory peptides have received substantial research, and some of these peptides, including IPP, VPP, IPA, PP, and GKP, have an antihypertensive activity [32,33]. According to previous reports, antihypertensive peptides are used as a successful therapeutic candidate for people with moderate hypertension or even a supplementary treatment [34]. Similar to this, the antihypertensive effects of IGLF and RVPSL peptides from egg white have been shown to reduce SBP in SHR rats [35].

Functional bioactive peptides with small amino acid sequences could successfully sustain bodily functioning and treat or prevent disease. Bioactive peptides isolated from soybeans have been demonstrated to function as an antihypertensive drug by lowering ACE activity [36]. Soy peptides that had been enzyme-hydrolyzed promoted endothelium-independent vasorelaxation and reduced ACE activity [37]. ACE-inhibitory peptides were also produced by alcalase hydrolysates of soybeans, which showed strong ACE inhibition [15]. VHVV bioactive peptide from soybean protects the liver and skeletal muscle of the animals fed with a high-fat diet [22]. The soybean bioactive peptide VHVV, when taken as a supplement, exhibits multiorgan protection, excellent antidiabetic action, and minimizes hyperglycemia-induced organ damage [38]. Soybean peptide VHVV also prevents long-term memory loss induced by hypertension. Animals treated with the bioactive peptide VHVV increased the expression of proteins associated with long-term memory and that promote neuronal survival. Oral treatment of the bioactive peptide VHVV activated CREB-mediated downstream proteins in spontaneously hypertensive rats, perhaps preventing long-term memory loss and maintaining neuronal survival [23].

Most of the endogenous ROS in cells are produced by mitochondria, and these hyper-generated ROS in the cells induce apoptosis. According to previous reports, mitochondrial dysfunction also results in hypertensive myocardial injury [39]. Mitochondrial fusion and fission control mitochondrial homeostasis in the heart under normal circumstances. However, when under stress, mitochondrial fusion promotes the repair of damaged mitochondria as well as the regeneration of new mitochondria. Contrarily, mitophagy eliminates damaged mitochondria by mitochondrial fission, but excessive mitochondrial fission under hypertensive circumstances causes apoptotic cell death and tissue fibrosis [40,41,42].

AMPK signaling and increasing glucose metabolism have been the main targets for developing therapeutic drugs for hypertension. In hypertensive rats, treatment with ACE inhibitor increases AMPK phosphorylation, which in turn prevents hypertension [43]. As a result, activating AMPK signaling might be seen as a protective strategy in the treatment of hypertension [44]. Exercise and mitochondrial-derived peptide (MOTS-c) are combined to potentially treat hypertension. When AMPK is stimulated, the MOTS-c can target the folate cycle. The MOTS-c expression is decreased by the downregulation of PGC-1α. Expression of MOTS-c is decreased by the downregulation of PGC-1α. Additionally, increasing PGC-1α and MOTS-c levels is a benefit of treadmill exercise training. Exercise and MOTS-c work in combination to increase PGC-1α expression through AMPK phosphorylation [45]. Hypertension can be reduced by using preventative substances that target AMPK signaling.

Cellular energy metabolism is tightly regulated in cardiac tissue through AMPK/SIRT/PGC-1α signaling pathway. PGC-1α, a nuclear transcription cofactor, can enhance mitochondrial production, lower insulin resistance, and control lipid metabolism [46]. PGC-1α protects the myocardium and plays a key role in regulating hypertrophy. When the ratio of AMP to ATP rises under glucose deprivation conditions or in response to pathologic stimuli, AMPK, which controls cellular energy balance, is activated. AMPK could control metabolic adaptations such as glucose transport and lipid oxidation [47]. In addition, AMPK may directly phosphorylate PGC-1α to activate it. The sirtuin family, which consists of SIRT1, SIRT2, SIRT3, SIRT4, SIRT5, SIRT6, and SIRT7, has been implicated in a variety of biological functions, including cell division, proliferation, and apoptosis as well as mitochondrial activity, energy metabolism, and longevity. SIRT1 and SIRT3 have been shown to enhance mitochondrial activity by deacetylating and activating PGC-1α [48].

Running, cycling, or swimming at a moderate effort for 30 to 45 min is the typical duration of aerobic activity. Exercise intensity is as significant an element in an exercise program as exercise frequency (the number of sessions per week) and duration (the length of time for each session) [49]. There is still a chance that too much exercise with insufficient recovery time might tilt the scales in the direction of negative consequences, given that an acute bout of exercise induces transient physiological stress [50]. Daily intense exercise increased mortality risk in individuals with evident coronary heart disease (on par with exercising just 1–4 times/month), and this was irrespective of several variables, including the presence of hypertension [51]. In our present study, animals have trained to swim for 20, 40, and 60 min for the first, second, and third week, respectively, which is moderate with health benefits.

In conclusion, our results substantiate that treating SHR animals with combined bioactive peptide VH-4 and swimming exercise training reduces hypertension synergistically. Both bioactive peptide VH-4 and exercise training alleviate hypertension-induced histological changes, activating PI3K/AKT cell survival pathway and AMPKα1, Sirt1, PGC1α, and FoX3α pathway.

## 4. Materials and Methods

### 4.1. Animal Grouping and Treatment

Wistar Kyoto rats (WKY) and SHR rats that were 12 weeks old were supplied by BioLasco Co., Ltd. (Taipei, Taiwan). Bioactive peptide VH-4 (VHVV (V—valine; H—histidine; V—valine; V—valine) was purchased from DG Peptides Co., Ltd. (Hangzhou, Zhejiang, China). The rats were maintained in 12 h light/dark cycles at a constant temperature of 22 °C and fed standard rat food and water. After the acclimation period, the animals were divided into five groups; SHR rats were randomly assigned to treatment groups (*n* = 8): Group I—WKY rats, Group II—SHR, Group III—SHREX, Group IV—SHRVH-4, and Group V—SHRVH-4EX.

VH4 (25 mg/kg BW) peptide was dissolved in PBS and administered intra-peritoneally for eight weeks. We chose this dose and administration route based on our previous reports [22,38,52]. For swimming exercise training, the rats were allowed to swim freely in a plastic tank with a diameter of 45 cm and warm water (35 ± 2 °C) filled at the height of 50 cm. For exercise training, animals were allowed to swim freely. Animals were trained to swim for 20, 40, and 60 min in the 1st, 2nd, and 3rd week, respectively; from the 4th to the 8th week, the animals were allowed to swim for 60 min. After the treatment period, the animals were killed, and the serum was separated from the collected blood. The animals’ hearts were dissected and cleaned with ice-cold phosphate-buffered saline (PBS). The serum and the heart tissues were kept at −80 °C until analysis. The Institutional Animal Care and Use Committee (IACUC) of Tunghai University in Taiwan, IACUC-107-40, gave its approval to animal experiments.

### 4.2. Blood Pressure Analysis

All blood pressure parameters in the treatment groups were measured by the tail-cuff method using a noninvasive blood pressure measurement system using Softron, BP-2010 series instrument (Softron Biotechnology Ltd., Tokyo, Japan).

### 4.3. Tissue Staining

Dissected heart tissue was washed in ice-cold saline and stored in 10% formalin. For preparing tissue sections, the formalin-stored tissue sample was washed in PBS, dried, and molded with paraffin. Thin tissue sections (about 2 μm) were prepared using a microtome and mounted on the glass slides. The paraffin wax was removed by incubating slides in a hot air oven. Slides with heart tissue sectioned were hydrated using graded alcohol and stained with hematoxylin and eosin (H&E) stain. The slides were then washed with PBS, dehydrated with graded alcohol, and covered using coverslips. The slides were observed under a microscope, and images were captured.

### 4.4. Western Blotting Analysis

Western blotting was performed using previously published techniques [53]. Briefly, 100 mg of heart tissue sample was homogenized in 1 mL of lysis buffer in ice and centrifuged at 12,500 RPM for 10 min at 4 °C. Supernatant was collected, and protein concentration was quantified in each sample. About (25–40 µg) of protein was subjected to SDS-PAGE electrophoresis for 45–50 min and transferred to the PVDF membrane using western blot apparatus (Bio-Rad Laboratories, Hercules, CA, USA). PVDF membrane was then incubated with 5% skim milk powder in TBST for 60 min and washed with TBS thrice. Then, the PVDF membrane was incubated with a specific primary antibody (1:1000 dilution in TBST) overnight at 4 °C and washed with TBS thrice. The membrane was then incubated with the corresponding secondary antibody (1:5000 dilution in TBST) at room temperature for 60 min and washed with TBS thrice. Finally, the protein of interest was visualized and documented using chemiluminescence ECL solution under Chemi-Doc apparatus (Fuji-film LAS-3000, GE Healthcare, Chicago, IL, USA).

### 4.5. Statistical Analysis

Statistical analysis was conducted using one-way ANOVA followed by Tukey’s multiple comparison tests. SPSS (SPSS, Inc., Chicago, IL, USA) software was used to process the data. The results are expressed as mean ± SD of three independent experiments.

## Figures and Tables

**Table 1:** Effect of VH-4 bioactive peptides and exercise on hemodynamic parameters.

BP	WKY	SHR	SHREX	SHRVH-4	SHRVH-4EX
HR	328.8 ± 14.70	423.0 ± 5.16 #	357.6 ± 14.69 ***	380.67 ± 9.79 ***	358.0 ± 7.55 ***
SBP	147.3 ± 12.09	212.0 ± 7.48 #	187.6 ± 8.50	189 ± 7.68	192.3 ± 22.50
MBP	116.6 ± 5.13	188.6 ± 9.46 #	147.0 ± 2.00 *	158 ± 6.22 *	149.6 ± 14.43 *
DBP	106.0 ± 5.29	175.3 ± 8.95 #	126.6 ± 2.51 **	142 ± 7.99 **	116.0 ± 12.52 ***

BP—blood pressure, HR—Heart rate, SBP—systolic blood pressure, MBP—mean blood pressure, DBP—diastolic blood pressure. # *p* < 0.05 compared to WKY, * *p* < 0.05, ** *p* < 0.01, and *** *p* < 0.001 compared to SHR.

## Data Availability

The data that support the findings of this study are available on request from the corresponding author.
